# Editorial: Glycocalyx in physiology and vascular related diseases–volume II

**DOI:** 10.3389/fcell.2024.1471251

**Published:** 2024-08-08

**Authors:** Ye Zeng, Bingmei M. Fu

**Affiliations:** ^1^ Institute of Biomedical Engineering, West China School of Basic Medical Sciences and Forensic Medicine, Sichuan University, Chengdu, China; ^2^ Department of Biomedical Engineering, The City College of the City University of New York, New York, NY, United States

**Keywords:** glycocalyx, homeostasis, dysfunction, cell response, molecular mechanism

## Introduction

The glycocalyx, a complex mesh of glycoproteins and polysaccharides enveloping the surface of various cells, has emerged as a pivotal element in both health and disease. This ubiquitous structure is not merely a passive barrier but a dynamic participant in numerous physiological processes and a critical mediator in pathological states. Insights gleaned from recent studies underscore the potential of the glycocalyx as a therapeutic target, promising new avenues for treating a spectrum of vascular-related diseases.

In this Research Topic, the glycocalyx is investigated in various contexts, including atherosclerosis, bladder cancer, inflammation, hypertension, sepsis, and trauma. It highlights the importance of understanding the mechanisms by which the structure and composition of the glycocalyx are modified, particularly in response to pathological changes.

### Blood flow and endothelial functionality

Zhou et al. demonstrate the pivotal role of blood flow-induced mechanical forces, particularly fluid shear stress, in regulating endothelial functionality. Their review emphasizes the interaction between blood flow patterns and endothelial cells, highlighting the significance of the endothelial glycocalyx in maintaining vascular homeostasis. The configurational changes of the glycocalyx in response to shear stress are crucial for understanding the role of endothelial cells in vascular functions.

Vittum et al. delve into the response to the shear stress by the basal endothelial glycocalyx. The review distinguishes between the apical and basal glycocalyx, each exposed to different stimuli, and explores their respective roles in vascular homeostasis. Understanding how the basal glycocalyx responds to fluid shear stress and its implications in pathologies offers a comprehensive view of endothelial glycocalyx functions.

### Glycocalyx in cancer progression

In the realm of oncology, Enomoto et al. focus on the glycocalyx’s role in bladder cancer. They utilize imaging techniques to visualize the aberrant glycocalyx in cancers, particularly invasive urothelial carcinomas. The distinctive staining of Vicia villosa lectin (VVL) in these carcinomas suggests its potential as a marker for cancer invasiveness. These findings underscore the glycocalyx’s involvement in cancer progression and highlight VVL as a promising target for future drug development strategies.

### Inflammation and coagulation

Ferreira et al. explore the endothelial glycocalyx’s involvement in inflammation-induced coagulation. The interplay between the glycocalyx, von Willebrand factor, and P-selectin is critical for understanding coagulation mechanisms during inflammation. They highlight that degradation of the glycocalyx leaves P-selectin as the sole anchor for von Willebrand factor, potentially leading to coagulation through unknown molecular mechanisms.

### Impact of high salt and HIV

Masenga et al. discuss the detrimental effects of high salt intake and HIV infection on the endothelial glycocalyx, contributing to salt-sensitive hypertension. The study emphasizes the synergistic impact of these factors in exacerbating endothelial dysfunction and hypertension. This research underscores the need for managing dietary salt intake and HIV treatment to protect glycocalyx integrity and prevent cardiovascular diseases.

### Trauma and glycocalyx modification

Richter et al. investigate the impact of trauma on heparan sulfate modifications within the endothelial glycocalyx. Trauma-induced glycocalyx shedding disrupts endothelial cell signaling, leading to pathological responses. The study identifies heparanase activity as a key player in glycocalyx degradation and subsequent endothelial dysfunction. These findings are crucial for developing strategies to mitigate trauma-induced vascular injuries.

### Atherosclerosis and syndecan-1

Qiu et al. highlight the role of syndecan-1, a vital component of the glycocalyx, as a predictor of plaque vulnerability in atherosclerosis. Elevated serum levels of syndecan-1 correlate with increased plaque vulnerability, suggesting its potential as a biomarker for cardiovascular risk assessment. This study provides a pathway for early identification and targeted treatment of atherosclerosis. However, further investigation is needed to fully understand the role and underlying mechanisms of syndecan-1 enrichment in advanced atherosclerotic plaques.

### Renal injury and brain death

Idouz et al. explore the cascading renal injury following brain death and the protective role of tacrolimus (FK506). The study demonstrates that tacrolimus preserves glycocalyx integrity and mitigates renal injury, highlighting its therapeutic potential in transplantation settings. This research emphasizes the importance of maintaining glycocalyx health in preventing renal dysfunction post brain death.

## Conclusion

The endothelial glycocalyx is a multifaceted structure that acts as a dynamic regulator in various physiological and pathological processes. From regulating blood flow and endothelial functionality to influencing atherosclerosis and cancer progression and to managing inflammation, the glycocalyx is integral to maintaining vascular health. Understanding its complex interactions and responses opens new therapeutic avenues for treating a wide range of diseases. As research progresses, targeting critical glycocalyx components, such as glycosaminoglycans and proteoglycans, could revolutionize medical treatments, providing more effective and personalized healthcare solutions.

